# Second opinion opportunity declined: patient typology and experiences regarding the decision-making process preceding elective surgeries in Germany

**DOI:** 10.1186/s12913-022-08742-4

**Published:** 2022-11-08

**Authors:** Susann May, Dunja Bruch, Felix Muehlensiepen, Barbara Prediger, Dawid Pieper, Cecile Ronckers, Sebastian von Peter, Edmund Neugebauer

**Affiliations:** 1 Brandenburg Center for Health Services Research, Brandenburg Medical School Theodor Fontane, Seebad 82/83, 15562 Rüdersdorf, Germany; 2 Faculty for Health Sciences Brandenburg, Brandenburg Medical School Theodor Fontane, 16816 Neuruppin, Germany; 3 Department of Cardiovascular Surgery, Heart Center Brandenburg, Brandenburg Medical School Theodor Fontane, Neuruppin, Germany; 4 Institute for Research in Operative Medicine, Witten/ Herdecke University, Witten, Germany; 5 Department of Health Services Research, School of Medicine, and Health Sciences, Carl von Ossietzky Universität Oldenburg, 26129 Oldenburg, Germany; 6 Brandenburg Medical School (Theodor Fontane), 16816 Neuruppin, Germany

**Keywords:** Second opinion, Decision-making, Elective surgery, Patient orientation, Shared decision making

## Abstract

**Background:**

To address the problem of overuse of elective surgery and to support patients in their decision-making process, a Second Opinion Directive was introduced in Germany, which enables patients with statutory health insurance to obtain a second opinion for certain surgical indications. The study aims to identify, based on the experiences of patients who have undergone elective surgery, the role of seeking a second opinion in reaching their decision.

**Methods:**

Sixty-two patients who had undergone an elective surgery (hysterectomy, tonsillectomy, shoulder arthroscopy) were recruited using purposive sampling and interviewed during October to December 2020. The transcribed interviews were analysed using a framework analysis to create a typology from the patient’s perspective.

**Results:**

The time spent by patients in making the decision to undergo surgery varies between individuals, and is influenced by factors such as the type of physician-patient relationship, individual patient aspects, prior experiences in the health care system, as well as information needs. Within the framework of the analysis, we were able to identify three patterns of patient types based on the three different time-points or phases when decisions were typically made, with one type being divided into two subtypes: Type 1a: Quick decision making, Type 1b: Overwhelmed quick decision making, Type 2: Time to consider, Type 3: Struggling with the decision.

**Conclusions:**

Patients who followed a recommendation for elective surgery appreciate having the possibility to seek a second opinion. However, various factors influenced their opting for a second opinion during the decision-making process. Patients have differing information needs, such that a one-size-fits-all second opinion service may not fit adequately for all patients.

**Supplementary Information:**

The online version contains supplementary material available at 10.1186/s12913-022-08742-4.

## Background

Health systems face the continuing challenge of providing adequate care, while striking a balance with neither underuse nor overuse. In particular, overuse of medical care is increasingly recognized around the world. Overuse refers to the provision of medical services for which the financial costs and the potential for harm to patients exceeds the potential for benefit [1, 2]. In this overuse scenario, a large number of patients receive medical services that bring them little or no benefit, and might better have been avoided [2, 3]. In Germany the so-called Second Opinion Directive (SOD) was introduced in December of 2018 to address overuse or unnecessary use of medical interventions. The first aim of the directive was to reduce the number of unnecessary elective surgeries [4]. Therefore, patients enrolled in statutory health insurance plans were entitled to obtain an independent and cost-free second opinion, in the specific cases of indication for elective hysterectomy, tonsillectomy, or tonsillotomy^1^ is given [4]. In 2020, shoulder arthroscopy procedures were added to the directive [5]^2^.^3^The conditions for which these types of surgery are indicated often bring long-standing, sometimes severe and debilitating symptoms, which were unresponsive to non-surgical interventions; accordingly, these conditions, while not life-threatening, bring a substantial deleterious impact on daily life and well-being. Main selection criteria for the four eligible surgical indications currently regulated by the SOD were the volumes of performed surgeries, regional practice variation in Germany, as well as the international experience^4^ [6]. A physician who provides a recommendation for SOD-covered types of surgery must inform the patients at least 10 days before planned surgery that they have the right to obtain a second opinion. When informing the patient about the right to obtain a second opinion, he or she has various duties to inform the patient. Supplementary file 1 contains information on the procedures of the SOD. Further information about the requirements for physicians who give the indication and those who wish to serve as second opinion provider can be found in May et al. [7]. Patients have the freedom to choose whether or not to seek a second opinion after being informed of their right to do so.

Of note, the German health care system historically includes generic opportunities for patients seeking a second opinion. This is enabled via structured programs developed by health insurance companies or by seeking advice from another physician, reflecting the free choice of physicians by patients, a pillar of German health care [8]. This kind of second opinion is referred to as an informal second opinion.

The second aim of the SOD is to support patients in their decision-making process. Making an informed decision to undergo surgery requires having detailed information about the various therapy options, which include conservative strategies without intervention (e.g., watchful waiting), for due consideration of their effectiveness and potential risks.

Coming to a decision in such matters can be very stressful for patients. Thus, Krohne et al. reported that patients often experience surgery as being something unpredictable and uncontrollable. Patients express worries about surgical or anesthesiologic complications, post-operative pain, or possibly unmanageable consequences that might arise after the treatment [9]. The decision to undergo a surgery or not requires detailed information about different therapy options concerning their effectiveness and side effects. Out of this need patients often move away from paternalism towards more equitable and collaborative models of healthcare delivery [10, 11]. Nowadays, when deciding for or against surgery, it can be helpful for some patients to get a second opinion to meet their information needs.

In general, there is a high demand for second opinions regarding diagnostic or treatment options in Germany regardless of the SOD [12]. Geraedts & Kraska surveyed 1598 people across the country, finding more than two-thirds of respondents thought it important that the health system should offer the possibility to get a second opinion, which they held as relevant and useful for their decision-making process [12]. Regarding patient characteristics and motivational aspects, previous studies from other countries have demonstrated that women tend to seek a second opinion more often than men, and that most patients who seek a second opinion are middle-aged and have a relatively high level of education [13]. The primary motivations of patients in seeking a second opinion are their need for certainty about diagnosis or treatment options, a lack of trust in practitioners, dissatisfaction with the level of communication, or a need for more detailed information [14, 15]. However, there is currently no exploration in depth of why certain patients decline a second opinion and the role of seeking a second opinion in patients’ overall decision-making process to undergo elective forms of surgery has yet to be explored.

The present study is part of the ZWEIT-project [8], which examines the characteristics and use of second opinion programs in Germany, as well as considering the resulting needs and wishes from the perspectives of physicians [7, 16] and of patients [17–19]. Here we focus on patients’ decision-making process, aiming to identify needs when deciding for or against an elective surgery, as well as factors that influence the decision to seek or decline a second opinion. In the spirit of the demand expressed by Greenfield et al.: “*Research […] is needed to identify patient groups that are likely to benefit from a second opinion*” [13], we now aim to develop a theoretical framework, independent of the directive, for recording the perceptions and experiences of patients who have undergone an elective surgery, and to identify the individual factors leading to a request for second opinion, which could allow tailoring of structured second opinion programs according to individual needs. In this context, the overarching aim of this study is to generate a patient typology in relation to the decision-making process that might identify which aspects influence the seeking of a second opinion prior to elective surgery.

Research questions:

- How do patients experience the process of decision-making for an elective surgery?
- How important is seeking a second opinion in this decision-making process?
- What types of decision-making behaviours can be identified?

## Methods

### Study design

This paper reports on our exploration of the decision-making process from the patients’ perspective, in their deliberations about undergoing an elective surgery, and utilizing their option to obtain a second opinion. We conducted in-depth interviews and used a framework analysis [20, 21]. The overall objective of framework analysis is to identify, describe, and interpret key patterns within and across cases of the phenomenon of interest, and its component themes [22]. Establishing such a typology could help researchers and stakeholders in the health system to understand the factors influencing patients’ medical decision making, and therefore we used framework analysis to create a typology from patients’ perspectives.

This study was approved by the Ethics Committee of the Brandenburg Medical School Theodor Fontane (E-01-20190529). Written consent was obtained after participants were given the opportunity to ask clarifying questions. Patients were not involved in designing this study.

### Participants

Participants were selected using purposive sampling [23]. We included patients who had undergone elective tonsillectomy (TE), hysterectomy (HE), or shoulder arthroscopy (SA) for non-malignant underlying diseases. In alignment with the eligibility criteria for the SOD [4, 8], patients with malignant diseases were excluded because multidisciplinary oncological care services (e.g., tumor conferences) are widely implemented in Germany [24]. Patients with a tonsillotomy were excluded because this procedure is predominantly performed on underage children and children are excluded from the sample. Although the inclusion criteria for this study were based on the directive, it was not a criterion that the indication for surgery was given when the directive was already in force.

Patients were recruited via AOK Nordost, a statutory health insurer operating in the German federal states of Berlin, Brandenburg, and Mecklenburg-Western Pomerania, and covering approximately 1.76 million insured citizens. In October and November 2020, insured persons of AOK Nordost who received a TE/TT (*N* = 2288), HE (*N* = 2934) or SA (*N* = 3640) in 2018, 2019 and 2020 were contacted. Eligible individuals received an invitation by post in October 2020 from AOK Nordost to participate in the interviews, with follow-up postal contact after 2 weeks. At the same time as being invited to the interview, people were asked to participate in a questionnaire survey. Patients who desired to participate in an interview had the option of contacting study researchers by phone or via a digital contact form. AOK Nordost did not receive notices of participation, nor any information provided by the patients in the interviews. Patients were offered €20.00 as an incentive for their participation in the study. Socio-demographic data was requested in a telephone conversation even before the interview was conducted to ensure that the sample was as heterogeneous as possible. The interview sample have been selected according to the maximum variation criterion [25] and the following aspects were addressed: indication, age, sex, educational level, settlement pattern.

### Data collection

A preliminary semi-structured interview guide was drafted by a multiprofessional team (SM, DB, SvP). The semi-structured interview guide consisted of open-ended questions that explored how and when patients made their decision about surgery, what sources of information they used, who influenced or assisted with their decision, whether they sought a second opinion, and their reasons for that decision. Sample interview questions included: *“Can you please describe how you made the decision to have a surgery?”, “Would you please tell me when you made the decision to have your surgery?”, “Have you sought a second opinion, and if so, why?”* (please refer to Supplementary material 2 interview guide)*.* In addition, socio-demographic data were collected, including gender, age, settlement pattern (cities, towns, suburbs, and rural areas), educational level, and occupational qualification. To ensure clarity and relevance of the questions, we conducted a pilot test of the interview guide with help from five eligible patients recruited from clinics and outpatient physicians in the study’s catchment area. We adapted the study protocol [8] in view of severe restrictions imposed by the Covid-19 pandemic; accordingly, all interviews were conducted via telephone during October to December 2020. The interviews were recorded and transcribed verbatim. Data collection continued until no substantially new findings emerged and saturation of content was reached. Saturation of content is defined as code saturation, when no additional issues are identified and meaning saturation, when no further dimensions, nuances, or insights of issues can be found [26].

### Data analysis

**Fig. 1:**
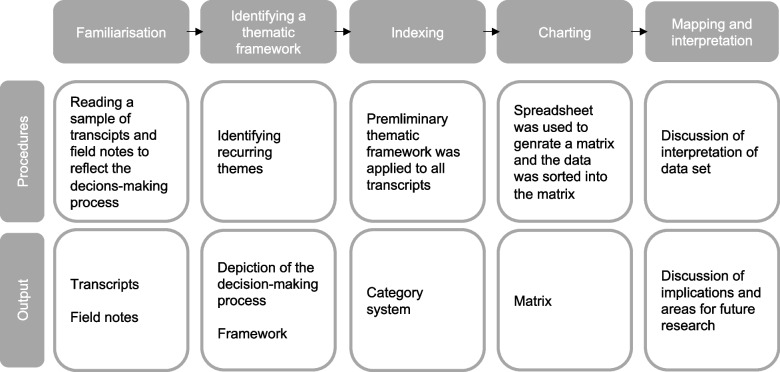
Study design and data analysis

Data collection and analysis based on Framework analysis [18, 19] were conducted with primary responsibility assigned to one researcher (SM) and with the assistance of the research team (DB, FM). Further information on data analysis is illustrated in Fig. 1. The transcriptions were made by a student assistant according to transcription rules [27]. After transcription of the audio material, the analysis began with a familiarization with the interviews, whereupon the interviews were coded (SM, DB, FM). Field notes consisted of a short summary of the interviews. The field notes were used to ensure better comprehensibility of the interview situation. The field notes themselves were not included in the analysis. Recurring main themes were noted and collated into groups of similar themes (SM, DB, FM). These groups were later organized into a draft theoretical framework or index, which was refined upon discussion among the team members (SM, DB, FM). We used thematic coding to develop our decision-making typology, focusing on key elements of participants’ experiences and major factors influencing a decision in favor of undergoing a surgery, and the role of seeking a second opinion in opting for surgery (SM, DB, FM). We systematically applied the analytical framework to every interview (SM). In this study phase, the themes were revised or merged, with formation of new categories as necessary. After revision, we summarized the data in thematic charts in an Excel spreadsheet (SM). In the final phase, all team members met to map, interpret, and make a synthesis of the data through reviewing the charts. We created a schematic diagram representing a typology of the experienced decision-making process in the context of seeking a second opinion, with grouping of similar themes based on the best fit to the data. We used MAXQDA software (Verbi GmbH) for our initial thematic coding, followed by Microsoft Excel (2021, Version 16.55) for our charting data. This manuscript has been compiled in accordance with the Consolidated Criteria for Reporting Qualitative Research (COREQ) [28] (please refer to Supplementary Material 3). For the presentation of the results, we have selected representative quotes of the discussion transcript for translation into English and inclusion in the text.

## Results

A total of 150 individuals have reported back, not all of whom were eligible for participation according to the inclusion criteria. Reasons for exclusion were either existence of malignant disease (which is explicitely excluded from the SOD, i.e., the SOD does not concern patients with underlying malignant conditions) or insufficient knowledge of German. A total of 67 interviews were conducted, of which five had to be excluded: Four participants were excluded owing to a history of malignant disease, and another was excluded due to failure to adequately address the interview questions. Of the 62 interviews proceeding to analysis, 23 patients have had a HE, 18 patients have had a TE and 21 patients have had a SA. The mean age of the participants was 50 years. The interviews lasted between 24 and 43 min (mean 28). An overview of socio-demographic information is shown in Table 1. For detailed socio-demographic information please see Supplementary Material 4.

**Table 1:** Characteristics of participants in the ZWEIT Interview Study among individuals who had undergone elective types of surgery during 2018–2020

Characteristic	Participants (*n* = 62)			
	total *n* = 62	HE *n* = 23	TE *n* = 18	SA *n* = 21
Sex				
Male	19(31%)	0(0%)	8(44%)	11(52%)
Female	43(69%)	23(100%)	10(56%)	10(48%)
Age at interview, yrs.				
19–24	4(6%)	0(0%)	4(22%)	0(0%)
25–34	7(11%)	1(4%)	6(33%)	0(0%)
35–44	13(21%)	6(26%)	5(28%)	2(10%)
45–54	12(19%)	10(43%)	1(6%)	1(5%)
55–64	14(23%)	2(9%)	1(6%)	11(52%)
65 or older	12(19%)	4(17%)	1(6%)	7(33%)
Educational level				
No formal schooling	2(3%)	0(0%)	1(6%)	1(5%)
9–10 years of school completed	45(73%)	18(57%)	9(50%)	18(86%)
> 10 years of school completed	15(24%)	5(22%)	8(44%)	2(10%)
Highest qualification				
None	5(8%)	0(0%)	3(17%)	2(10%)
Apprenticeship	48(77%)	20(87%)	12(67%)	16(76%)
Bachelors	2(3%)	1(4%)	0(0%)	1(5%)
Masters or Magisters degree/ Diploma	7(11%)	2(9%)	3(17%)	2(10%)
Settlement pattern (Number of inhabitants)				
Rural region (< 5.000)	8(13%)	4(17%)	0(0%)	4(19%)
Towns and suburbs (> 5.000–100.000)	17(27%)	7(30%)	5(28%)	5(24%)
Cities (> 100.000)	37(60%)	12(52%)	13(72%)	12(57%)
Obtained a second Opinion^a^				
Yes	8(13%)	4(17%)	1(6%)	3(14%9
No	54(87%)	19(83%)	17(94%)	18(86%)

Abbreviations: *HE* Hysterectomy, *TE* Tonsillectomy, *SA* Shoulder Arthroscopy

^a^Based on self-report by the interviewee; this variable refers to having sought a second opinion specifically related to the symptoms and indication for the respective surgery that defined eligibility for this research study

### Depiction of the decision-making process as experienced

Initially, we captured the timeline of the decision-making process in order to enable a detailed description of the process. Then, the potentially relevant motivational aspects that influenced seeking a second opinion were reported by the patients.

#### Time prior the indication

The specific indications for elective surgery captured by this study were preceded by a lengthy period of symptoms and management for each patient, generally characterised by functional limitations, pain, and suffering. The patients describe a subjectively perceived poor state of health with long-lasting symptoms associated with restrictions in their daily lives. The indications^5^ considered here were not usually received in the beginning of the treatment; the duration of symptoms prior to the indication could differ greatly between individuals.

*“I just wanted the pain to stop, and simply wanted to return to work and be free of pain at work or in my daily life.”* (MaG238_SA, Pos. 56).

#### Moment of indication

Most of the interviewees experienced the moment of indication in a similar manner: due to the persistence of their symptoms, and frequently due to unsuccessful results of alternative medical interventions, the moment when the physician indicated eligibility for surgery (i.e., the ‘Indication’) was frequently described as “relieving”, combined with joyful anticipation:

*“The referral to surgery was basically a ray of sunlight, a (moment of) relief.*” (MäG210_HE, Pos. 8.).

However, there were patients who experienced the moment of receiving the indication negatively. Such respondents reported that they felt under pressure from the attending physician during the indication process. They felt the lack of a comprehensive explanation, and that the recommendation for surgery was from their perspective premature.

*“I had no choice. “You just have to decide and that is that; I give you half an hour”. What can you do in half an hour! Then they tell what might happen, and that you cannot make any other decision. If you want to continue living, if you think of the kids, and don’t want to take any risks, then that’s the way it is. You must have trust in your doctors, and there is no other choice for you.”* (MaG228_HE, Pos. 26).

#### Moment and phases of decision-making

The time after referral when patients decided whether to consent to surgery differed between individuals. We identified three different time-points or phases when decisions were typically made. The first phase, during which a number of patients had decided to undergo surgery, was immediate, arising during the same conversation in which the physician provided the indication.

*“The doctor believed that he had to operate. I immediately said, OK, let’s go for it. He is the doctor. He knows what he is doing. He is the specialist.”* (MaG227_SA, Pos. 42).

A second group of patients did not decide immediately, but rather arrived at their final decision after a certain period of time, during which they informed themselves about their disease and therapy options and discussed the matter with family members or their social network. The period of time until arriving at such a decision could last from days to a few months.

*“So, I’ve always been one to weight things carefully. I reflect/consider. And, as the saying goes, «sleep on it». It took me several nights to come to a decision.”* (MaG220_SA, Pos. 44).

The remaining patients needed considerably more time to reach a decision, with a delay that could last for years in some cases. During this decision phase, all treatment alternatives had been exploited and surgery had become the last option. This phase is characterised by uncertainty and struggling with the decision.

*“Yes, well, it wasn’t that I’d decided straight away that I wanted the surgery. I think it was only two years later: more and more I’d resolved myself to this decision [over that time]. Before that, there was still a certain uncertainty, because of the risks, I’d also... Well, there are always risks associated with a surgery and that was still a bit on my mind and that’s why it was definitely a longer process before I made up my mind. Actually, I’d already started to collect evidence or proof for my decision earlier.”* (MaG247_TE, Pos 33).

### Depiction of factors influencing decision-making - motivational aspects behind seeking no second opinion

The patients were asked whether they had sought a second opinion during their decision-making process, and were offered the opportunity to elaborate upon their answer. In sum, 54 of 62 (87%) respondents had not sought a second opinion. We identified three overarching motivational dimensions that explained why people did or did not seek a second opinion: (1) patient-physician relationship; (2) patient intrinsic aspects; and (3) adverse experiences in the health care sector. These are elaborated upon below, and we present further factors that emerged as influencing decision-making for surgery.

Although not all patients in this study population had the right to seek a second opinion according to the directive, because the directive had not yet been implemented for all participants at the time of receiving the indication, the majority of participants appreciated the opportunity to seek a second opinion.

*“That’s where I would have immediately gone for a second opinion if my doctor or my gynaecologist had said, “Go there, please [to a second opinion physician].” Then I would have immediately sought a second opinion. Immediately.”* (MaG213_HE, Pos. 16).

#### Profound relationship between patient and physician

In analyzing the interviews, it became apparent that trust was an important element in the decision-making process. Patients with a trusting relationship with their physician did not generally consider seeking a second opinion. Patients often reported having a long-term relationship with the physician who provided the indication. Often the physician would know about previous illnesses and the current state of the patient’s disease, such that the patient felt understood and well treated.

*“Because I trust the orthopedists so very much [...] and I feel very well looked after at this medical practice. It never occurred to me to have a second opinion, which I know that I am in good hands.” *(MaG214_SA, Pos. 16).

Clear and complete communication was seen as an essential component of the quality of care provided. As long as physicians provided reliable information, listened attentively, and gave thorough explanations, seeking a second opinion was not considered.

*“[…] because I was simply satisfied that I had found a solution. I had the impression [at the doctor’s] that I was in the presence of a competent specialist who would listen to me, take me seriously, and explain everything to me.” *(MaG219_SA, Pos. 21).

Some patients reported that they did not feel sufficiently informed about the procedure and its consequences and would have wished for more information.

*“But I was not informed at all about what would happen. What it would be like after the surgery and what to expect. I was stunned that I was lying in bed like an idiot, with the splint and everything. It was terrible.”* (MäG203_SA, Pos. 26).

Other patients describe concern that seeking a second opinion would undermine the relationship of trust with the physician.

*“I have to say, however, that it’s not that easy to just get a second opinion, because it’s a little embarrassing and could be understood as distrust. And, well, I would have felt uneasy (telling the doctor that I’d like to get a second opinion.)”* (MaG222_HE, Pos. 87–89).

#### Patients’ intrinsic aspects

A high level of suffering was one of the main aspects that was mentioned in the interviews. Seeking a second opinion was not an option for most of the interviewees, because frequently they had been suffering for a long time. This is in line with patients not wanting waste time because they wish to quickly return to “normal life”.

*“So, I didn’t need a second opinion because I just want to bring the matter to a close, since, to be honest, I found it abnormal. Because it was so disgusting to leak like a bucket when I had a period. And I grew weary of that.” *(MaG206_HE, Pos. 44).

Often patients had developed a desire for surgery before its indication was provided, such that the physician only served to confirm their decision:

*“I want the surgery. And she agreed and immediately arranged my transfer to the hospital.”* (MäG213_HE, Pos. 8).

#### Adverse experiences of healthcare

Many participants mentioned past adverse experiences in the health care system. From the patients’ perspective, a mix of negative and sometimes plainly concerning encounters were recounted. Based on such prior experiences in the health care system, patients had preconceptions that they would need to wait a long time to obtain an appointment to be seen by a physician certified to give a second opinion.

*“I didn’t even think about it. You know, that was simply too time-consuming for me. You have to wait for months before getting an appointment. And here at the doctors it takes three months. I was quite happy with that.”* (MaG209_SA, Pos. 24).

In addition, some patients were concerned that even if they got an appointment promptly, they would have to wait for a long time in the practice waiting room. Obviously, waiting time goes hand in hand with an effort that the patients are unwilling to tolerate.

*“Interviewer: Have you considered getting a second opinion?*

*Participant: Nope. On the one hand, you probably think, yes, that is necessary. But you always sit in the doctor’s waiting room for hours on end.”* (MäG210_HE, Pos. 23–24).

### Depiction of factors influencing decision-making - motivational aspects in seeking a second opinion

Eight of 62 (13%) of participants obtained a second opinion. Of eight participants who sought a second opinion, five lived in towns/suburbs (8%) and three in large cities (5%). None of the 62 participants from rural areas sought a second opinion. Three interviewees changed their primary physicians in the course of their care, and five interviewees remained under treatment by the same physician who had given the indication, after having obtained a second opinion. The three patients who changed doctors beforehand were all dissatisfied with their treatment due to what they felt was inadequate communication.

#### Various interpretations of second opinion

It was particularly noticeable that patients interpreted obtaining a second opinion in different ways. While the second opinion was understood as a consultation with another independent physician who had not been involved in initial care, it became clear that other types of physician contacts were also interpreted as constituting a second opinion. For example, an earlier change of specialist was perceived as such by some patients.

*“Then I went to see a gynecologist, actually a man who I regularly saw, who relatively early suggested removal of my uterus. Unfortunately, that wasn’t so friendly/sympathetic, and it wasn’t explained to me. At that point, I switched gynecologists, and sought out a second opinion myself.” *(MaG202_HE, Pos. 2).

In addition, patients sometimes interpreted the pre-surgery discussion in the clinic as constituting a second opinion.

*“Basically, the [preoperative talk] was for me a second opinion, anyways. If you like.”* (MäG210_HE, Pos. 45).

Unlike those who did not seek a second opinion, the eight participants who did so also identified the physician-patient relationship as one of the most important factors in their decision. These patients stated that they had sought a second opinion because their own doctor did not offer sufficient information or was too hasty in recommending the surgery, or did not give them sufficient time.

*“I went to the orthopedist. He just told me, “Pills and physiotherapy”. I wasn’t satisfied with this. He did nothing. I didn’t trust him.”* (MaG227_SA, Pos. 16).

A further aspect here was the uncertainty regarding the decision. Obtaining a second opinion is intended to provide certainty in the decision-making process, to help the patient avoid making the wrong decision regarding their surgery.

*“And yes, the second opinion was very important for me. So that I wouldn’t make the wrong decision. And that’s why it was so very important for me.”* (MaG201_HE, Pos. 38).

### Depiction of factors influencing decision-making - seeking health care information

Our analysis of the interviews revealed further factors influencing decision-making for surgery, which were not directly mentioned in the context of seeking a second opinion. The participants reported that opinions and experiences of family and friends bore a substantial influence on their decision to undergo elective surgery. Various topics were discussed with other people, such as the possible impact of the surgery or the choice of hospital or surgeon.

*“Nowadays, before making a decision, people not only inform themselves about the doctor’s opinion, but also on the internet and get experiences from their friends/family/acquaintances. So that’s what I’ve already done.”* (MäG210_HE, pos. 73).

Furthermore, the Internet was stated by the participants as a source of information that influenced their decision. Among the different types of websites that were consulted, were clinic websites, general information health homepages, as well as forums reporting personal experiences.

*“Well, yes, I looked online. A bit of general information on the subject, “What can I expect of such a surgery?”, and also a few ratings and testimonials from other people, whom I didn’t know personally. So when I think about it again, it had already influenced my decision.” *(MaG240_TE, Pos. 32).

**Fig. 2:**
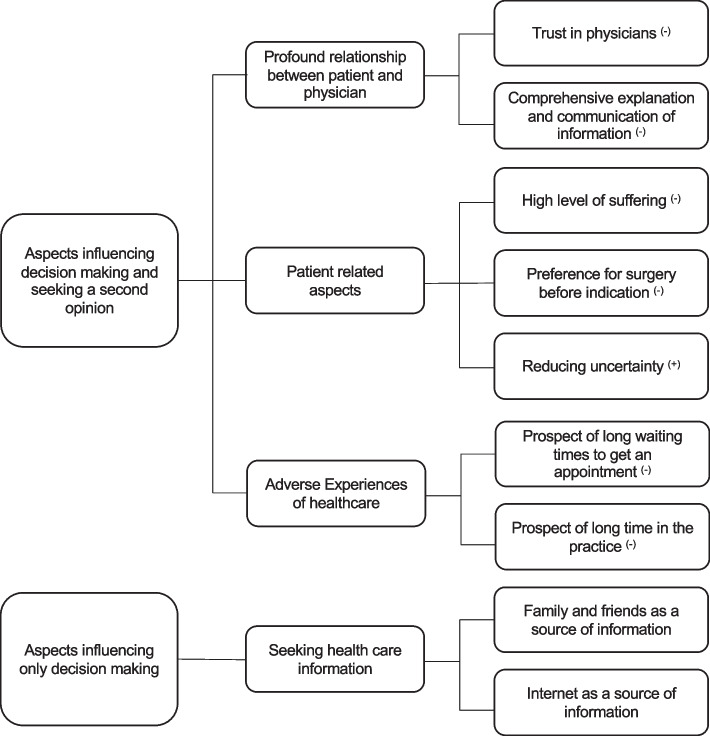
Category system of aspects influencing decision-making; (−) decreases the likelihood of someone seeking a second opinion, (+) increases the likelihood of someone seeking a second opinion

Figure 2 summarises the category system that describes the different motivational factors in the decision-making process of patients who had and had not sought a second opinion.

### Derivation of a typology of decision-making

**Fig. 3:**
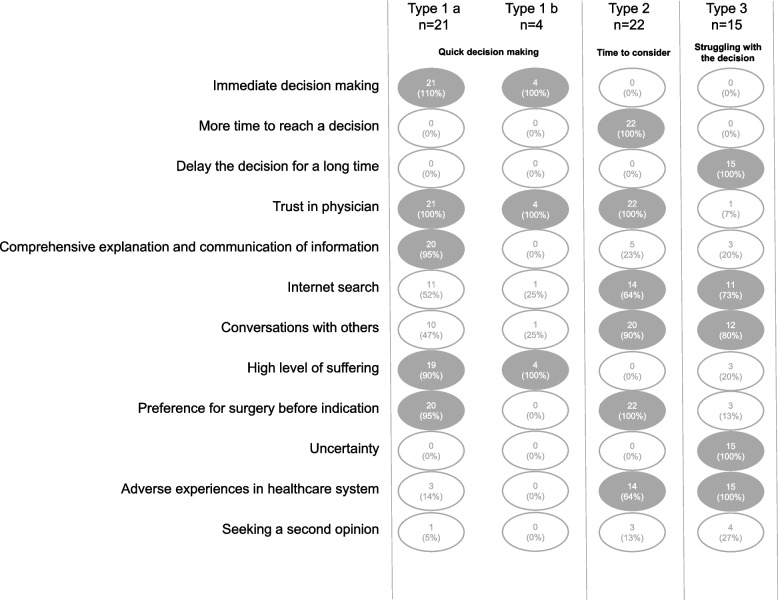
Typologymatrix

In the context of decision-making, we examined the temporal sequence from diagnosis to surgery, and were able to identify inductively the three time periods in which patients made their decision for or against surgery. For creating a typology, we assigned the patients to the three periods as described above: (1) the ‘immediate decision-making’, (2) the ‘more time to reach a decision-phase’, and (3) the ‘delay the decision for a long time-phase’. We then converted the motivational aspects of seeking a second opinion and the aspects influencing decision-making into variables. In order to enable a more reliable analysis, the category ‘Adverse experiences of healthcare’ was summarised by the composite of its subcategories. As a result, the three periods as described above and eight influencing factors were included in subsequent analysis, and the extent of their occurrence in the three typology groups was examined. Within the framework of the analysis, we were able to identify three patterns of patient types based on the three different time-points or phases when decisions were typically made. For one decision type, where the decision was made immediately, we could identify two subtypes (please see the matrix in Fig. 3). In the following we would like to illustrate the types with selected quotations.

### Type 1 a - quick decision making

This type makes the decision to undergo a surgery immediately. Type 1a is characterised by having great trust in the physician and feeling thoroughly informed.

*“The guy (the doctor) knows what he’s doing. At that point I didn’t think about a second opinion, I trusted him” *(MaG219_SA, Pos. 23).

*“Honestly, no. The information I’d gotten there was enough for me, actually. I had heard what I wanted to hear and well, I wanted to get it going. So, it was pretty clear for me at that point that I would do it (the surgery).”* (MaG237_TE, Pos. 28).

Patients associated with this type preferred surgery before indication.

*“Then I had already decided that it had to be done. That there was no alternative for me. Even before the doctor had brought up the surgery.”* (MaG207_SA, Pos. 40).

This type has a high level of suffering but is confident in decision-making.

*“Well, I couldn’t even raise my arm any more, and had no strength besides. I was suffering so much. And then I didn’t hesitate at all and said, “OK, then it has to be done, what’s the point of getting a second opinion?”* (MäG220_SA, Pos. 48).

Prior experiences in the health system did not influence this type’s decision to undergo a surgery. The probability that this type would seek a second opinion is low. In this group, one person (5%) has sought a second opinion.

### Type 1 b - overwhelmed quick decision making

This type also immediately decides to undergo a surgery. In contrast, this decision type is characterised by not feeling themselves sufficiently infomed, but still having a great deal of trust in the physician.

*“I had no choice […]. Then he tells you all the things that could happen, and that there’s no other decision. If you want to live, for the kids and all that, don’t want to take the risk, then it can’t go any other way. You’ve got to trust the doctors and then you’ve got nothing else.”* (MaG228_HE, Pos. 26).

*“Yes, I didn’t have a lot of time then. Well, I’d felt that I was completely abandoned to deal with everything. That I couldn’t make a decision at peace because I hadn’t been informed. I definitely felt that I was missing information and I just felt bad. Yes. And then to put your entire trust on the doctor again, that’s a pretty difficult thing.”* (MaG234_HE, Pos. 14).

This type also had a high level of suffering but did not consider surgery before the indication was made.

*“But I was in such awful pain that I couldn’t think things through for so long, because I just wanted to get clear of this situation with the strong decline, you know? And to be able to do sports again, and yeah. And then I decided to have the surgery. But as I said, it was such a shock for me, you know? And I thought: ‚That‘s pretty strange, that nobody properly explained all of this before.”* (MaG234_HE, Pos. 4).

*“Yes, um, my age was decisive, actually, and because of my age, it was explained to me that this would be a prophylactic opportunity to prevent, uh, possible cancer. Actually, that convinced me because, well, there was no need to absolutely keep one of my sexual organs. I’d never thought about a surgery before the doctor recommended it.”* (MaG222_HE, Pos. 4).

The prior experiences of this type in the health system did not influence the decision to undergo a surgery. The probability that these patients may regret the operation is believed to be comparatively high.

*“I think that that was the biggest mistake I’ve ever made. I absolutely regret it.” *(MaG228_HE, Pos. 60.

The probability that this type would seek a second opinion is low. No one in this group has sought a second opinion.

### Type 2 - time to consider

This type is characterised having a great of trust in the physician.

*“I definitely have a trusting relationship. Because before I’d already had complaints and could come any time. She always took time. I think that’s really good.”* (MaG217_HE, Pos. 21).

However, this type does not feel sufficiently informed by the physician.

*“Nah, no one told me anything (about treatment possibilities or the surgery). In general I didn’t feel so well informed.”* (MaG208_TE, Pos. 16).

The pressure of suffering was moderate. This type had also considered surgery before the indication was made, and was quite confident in their decision-making. This type researches online and shares information with others.

*“Basically, I make a decision for me, but I talked to my parents about it as well. Asked them if they also would see it the same way, having experienced what they’d experienced. That helped me with the decision […]. I googled how it works and what dangers might possibly arise. Those weren’t clear for me, from the doctor’s explanation. But it didn’t scare me away, in any case.” *(MaG229_TE, Pos. 26–32).

The probability of this type seeking a second opinion was comparatively medium. In this group, three people (13%) sought a second opinion. Adverse experiences in the healthcare system, such as the prospect of long waiting times, discourage these patients from seeking a second opinion.

### Type 3 - struggling with the decision

This decision type is characterised by a lack of trust in the physician and feeling not sufficiently informed.

*“Nah, I honestly don’t feel well-taken-care-of […]. And I thought, like: Well, OK, she’s nearby, I can get there quickly, I’ll just stay there. Well, nah, I don’t particularly trust them.” *(MaG221_HE, Pos. 35).

*“I don’t make it easy for myself [getting the surgery]. Well, I weigh the advantages and disadvantages, and try as much as I can to inform myself about the possible risks, what could then happen to me on the way if I did decide to do it, what kinds of possibilities and options are available, and what difficulties and other hindrances could also happen to someone. Well, I didn’t get to know all of that properly from the doctor.”* (MaG246_TE, Pos. 39).

The pressure of suffering was not high, and this type had not considered surgery before indication. However, this type is uncertain, wants to avoid the surgery, and searches for information elsewhere.

*“When you hear ‘surgery’ for the first time, you don’t jump immediately at the opportunity, because you just don’t happily allow someone to go snipping around on you. At that point you’re absolutely uncertain. And for that reason I really wanted to hear what others were saying about it.”* (MaG233_HE, Pos. 28).

This type is influenced by their earlier adverse experiences with medical care. We expect that there is a higher probability that individuals of this type will seek a second opinion. In this group, four people (27%) sought a second opinion.

## Discussion

In this study we investigated the decision-making process for a set of elective surgeries as experienced by patients. As far as we know, this is the first study focusing on the decision-making process of elective surgeries in the context of obtaining a second opinion. In addition, we derived from our data a novel decision typology of people who have undergone elective surgery. With the help of this approach, we can derive criteria for successful counselling and information for patients, as well as actionable recommendations for an effective information and counselling procedure.

Prior studies have investigated patients who sought a second opinion, supporting a number of general conclusions about why a second opinion was sought. As an example, we note dissatisfaction with the primary care provider and insufficient provision of information and education [13, 15]. We have recapitulated those findings in our study. Furthermore, we have shown that individual patients have different needs for information, and regarding the entire decision-making process, and that an exchange with family and friends, as well as internet research, plays essential roles, as shown previously [29]. The most innovative elements of our study are that we focused on decision-making in the context of the second opinion and that we included respondents who had and had not opted for a second opinion. With this approach, we were able to add intrinsic factors and aspects related to the health system-related aspects to the factors already identified in other studies.

Obtaining a second opinion probably depends on the moment when the decision for surgery is made. Thus, the time window for patients to seek a second opinion varies depending on the decision-making type. We have identified several types of patients who may be more likely to benefit from the recommendation to seek a second opinion. Patients who feel well informed and have a trusting relationship with their physician are obviously less likely to seek a second opinion. These aspects can be observed in type 1 a. Thus, the interactions regarding the patient’s relationship with the referring physician seem to be the linchpin. However, it remains unknown whether this type of patient is really well-informed or whether this behaviour demonstrates a paternalistic physician-patient relationship. This type of patient may decide too quickly to undergo surgery and may not be aware of their own preferences. In this respect, it seems that in settings with an established physician-patient relationship, there is less interest in a second opinion. These patients were probably able to make a decision more quickly due to the subjectively perceived detailed explanation by and trust in the physician. In particular, trust and cooperation seem to be prerequisites for reaching a decision for or against a surgery, while mistrust and helplessness make this more difficult.

In contrast to type 1a, type 1b feels overwhelmed. We have only identified a small group that belongs to this type. Nevertheless, special attention should be focused on these patients, as they might regret the surgery afterwards. It is therefore a future challenge to identify the people who would presumably regret undergoing the surgery. It is important to give such patients sufficient time to make a decision, and not to make them feel obliged to undergo a surgery, by ensuring that their needs and concerns are not ignored [30].

Patients of type 2 (Time to consider) obviously have a higher need for information, and additionally inform themselves on the internet or exchange experiences and information with others. For these patients, it would be important to channel the need for information.

Particular attention should be focused on the group of patients (type 3) who are struggling with the decision at length. They may need our particular attention to anticipate possible difficulties in their participating in the treatment decision. This result is consistent with the findings of other studies that concluded that the time taken to decide increases with its difficulty [31], and that decisions with low conflict potential tend to be made faster, whereas decisions with high conflict potential are made more slowly [32]. It may be that patients who are struggling with their decision should not only be routinely informed about the possibility of seeking a second opinion, and/ or that physicians should offer them decision aids. A systematic review comprised of 115 controlled studies involving 34,444 participants reported that there is high-quality evidence that provision of decision aids compared to usual care improves a patient’s knowledge regarding options and relieves their decisional conflict [33]. The motivation for the introduction of the directive is particularly evident in this type of patient: on the one hand, to avoid unnecessary surgeries and, on the other hand, the second opinion can support patients who are uncertain in their decision-making. In this way, the aims of the SOD would be met. Especially in patients who take a long time to decide, the perceived threat seems to be higher due to bad prior experiences in the health care system, and may also have been influenced by unreliable internet sources and exchanges with other people.

In order not to put patients in conflict about whether or not to seek a second opinion, the shared decision-making (SDM) approach could be used as part of the initial treatment. SDM presents an opportunity for patients to be supported in the decision-making process. It is a collaborative process where clinicians and patients share the best available evidence when facing a decision [34, 35]. SDM reduces decision conflict and improves decision quality for patients making choices about elective surgery, and may influence patients to choose surgery less often [36]. A systematic review summarizes that SDM may promote a more positive healthcare experience and decision-making process for patients [36]. Thus, physicians should assess patients’ individual preferences and adjust the treatment recommendation accordingly. Studies suggest that in supporting patients to make decisions, physicians should not only provide medical information, but also talk to the patients to clarify their doubts about the surgery and to offer them whatever information they may need [37, 38].

Based on our results, patients appreciate being offered the opportunity to receive a second opinion, but still, in the end the majority of patients did not exercise their option to seek a second opinion. However, even if a physician offers that option, certain factors are important in determining whether or not it is used. In their analysis of the use of structured second opinion programs provided by German statutory and private health insurances, Könsgen et al. report a relatively low incidence of insured persons who make use of the second opinion programs [17]. This could perhaps reflect that not all patients expect any benefit from obtaining a second opinion.

The present results of our data-driven analysis can be discussed in the context of the Health Belief Model (HBM). The HBM assumes that the perceived threat of a disease triggers consequent action. The perceived threat is composed of the individual’s perception of their own susceptibility to disease and their belief that this disease may have serious consequences. The degree that a person believes a certain course of action, such as surgery, to be beneficial, and whether these benefits outweigh perceived barriers, determines the likelihood that a person will change their health behaviour [39].

An important aspect of our results is that patients’ experiences in the health care system may influence them in seeking a second opinion. Presumably, patients would be more likely to decline a second opinion if they feel they are already receiving inadequate treatment, or conversely, if they have had a bad experience with treatment in the past. This is in line with other study findings that suggest that the perception of a previous negative experience may influence subsequent health-care-seeking behaviors [40]. Some specific consequences of a negative health care experience include avoidance of or delays in seeking further health care [41]. These behavior changes have been associated with lower satisfaction with care and lower-quality relationships with care providers [42]. In our study, we were able to identify that those who had a lot of trust in the physician (type 1) were more likely to have had fewer bad experiences in the past and were also more likely not to have sought a second opinion. A previous qualitative study has also found that patients’ prior experiences with the health care system impact on their subsequent use of health services [43]. In this context, it should be certain that a sufficient number of approved second opinion physicians are indeed available, which can be subject to geographic constraints. The physician providing the indication should also inform patients that there will not be unduly long delays for an appointment with a second opinion physician. At this point, we note that that the geographic distribution of second opinion physicians in Germany varies from region to region, and is lower in rural settings [16]. Consequently, there can be some uncertainties in the waiting period, depending on the patient’s location.

Another important result of this study is the differing interpretations of the second opinion by patients. Obviously, there are some uncertainties in the exact definition of obtaining a second opinion. Here, there is a need for greater patient awareness throughout society and a strengthening of health literacy. Educating patients about the concept of a second opinion should not only be the responsibility of physicians. Rather, health insurance companies should be involved in this educational work, and national campaigns should be carried out. Educating patients that it is appropriate to seek a second opinion, especially when there is uncertainty in the decision-making process, can also reduce fears of consulting a second physician [15, 44]. The acceptance of the directive would increase, making it more likely that patients would “dare” to seek a second opinion.

In the context of the future realisation of the SOD in Germany, all patients should of course be informed about their right to obtain a second opinion. Information needs when deciding for or against surgery obviously differ, so that obtaining a second opinion probably does not seem necessary for all patients. In summary, for the selected indications in this study, a guideline-based treatment plan could often obviate the perceived need to obtain a second opinion, and patient-centred care with shared decision approaches would help to avoid overuse.

Particularly in diseases where surgery is an option, treatment should be patient-centred, with not only clinical knowledge being communicated by the physician. Rather, the exchange should be empathetic and inquiring. Above all, physicians need to be aware that patients have differing information needs. Especially for patients who make the decision quickly (type 1), the physician providing the indication for surgery should consider whether the decision was made too quickly. It is especially important not to put patients under pressure when making decisions and to give them time. Patients of types 2 and 3 have a greater need for information. Within treatment, physicians should specifically inquire about the extent to which information needs exist and respond to them in a targeted manner. Decisions aids or checklists can be offered here.

### Strength and limitations

To the best of our knowledge, we have performed a qualitative framework analysis and derived a patient typology of patients who have undergone an elective surgery in Germany. The qualitative interviews allowed for obtaining an in-depth understanding of the experiences and motivational factors in seeking a second opinion or not, although it should be noted that the sample size is relatively large for a qualitative study. However, there are certain limitations to our study. The results might be subject to a selection bias because only one health insurance company was utilized to recruit eligible individuals. On average, insurants of AOK Nordost have a lower level of education and lower socioeconomic status than the general population [45], as is reflected in the demographic characteristics of participants to our study. There may be a greater incidence of negative prior experiences of the health care system, because patients from rural areas have frequently been faced with a shortage of specialists. However, it should be noted that half of the respondents were from a city with over a million inhabitants. Furthermore, the sample was very heterogeneous and with different indications (TE, HE, SA). Furthermore, consistent with the segment of the population served by our project partner, the Health Insurance Company AOK Nordost, participants with a high education are underrepresented in our study. This might have an impact on the results. Finally, the specific type of elective surgeries under consideration were associated with underlying conditions and symptoms that typically represent a considerable burden to patient well-being and interfere with daily life over prolonged periods of time. Additionally, owing to the study design, we were unable to recruit any patients who had received an indication for surgery, but declined to undergo the procedure (with or without seeking a second opinion). Nevertheless, the indications investigated here are generally relevant to SOD for Germany. We concede that the decision typology was not established in relation with personality characteristics, which may have had an independent influence on decision-making behaviour. With this in mind, further research is necessary, whereby the directive is currently being evaluated [46].

### Further research

The typology presented here is a first theoretical approach that focuses on the decision-making behaviour, and respectively the use of second opinions to patients who are facing surgery. The results are currently not yet validated by quantitative methods and should be investigated in further studies. In this context, it would then be conceivable to develop a tool that can identify the need for second opinions. It is conceivable that the likelihood of seeking a second opinion differs for elective surgery that does not result in prolonged pain or restrictions on participation in daily life. For example, breast reconstruction. If the pressure of suffering is not so high, then the need for information and the handling of the decision-making process may be different. Further research is needed to find out whether the decision-making process differs. Additional studies with patients who do and do not undergo surgery after obtaining a second opinion would be needed to apply the typology to these groups. A further recommendation could also be that insurance companies code consultations for identification of second opinions to support future understanding. As the coding is done by the physicians, they also need to be instructed accordingly.

Further research should also focus on the conflict that patients face when considering whether or not to consult another physician, as this situation could potentially be conflictual for patients, due to fear of causing offense. Research should continue to explore how perceptions of previous negative health care experiences and information needs are associated.

## Conclusion

Patients who underwent elective surgery after a physician recommendation appreciate having the option to obtain a second opinion. However, factors such as the relationship with the physician and his/her provision of sufficient information, as well as patient-related aspects and prior experiences of the healthcare system, influence the choice to obtain a second opinion during the decision-making process. Patients have different information needs such that there is no one-size-fits-all second opinion service for all patients. Presumably, the SOD cannot holistically address the problem of overuse. Comparative studies are needed to determine whether SOD can curb overuse. Rather, future health care plans should be more patient-centred and focus on increasing health literacy.

## Supplementary Information

**Additional file 1.** Procedure of the SOD

**Additional file 2.** Interview guide

**Additional file 3.** COREQ.

**Additional file 4.** Detailed socio-demographic information

## Data Availability

All data relevant to the study are included in the article or uploaded as supplementary information. For further questions regarding the reuse of data, please contact the corresponding author (susann.may@mhb-fontane.de).

## Abbreviations

SOD: Second Opinion Directive
HE: Hysterectomy
TE: Tonsillectomy
TT: Tonsillotomy
SA: Shoulder Arthroscopy
SDM: Shared Decision Making
HBM: Health belief Model

## Footnotes

1 Excluded from the SOD are indications due to malignant diseases

2 Excluded from the SOD are indications due to accident-related causes

3 Other indications, such as implantation knee endoprosthesis and amputation for diabetic foot syndrome, were successively included in the SOD during the course of this project.

4 Based on a national and international literature search, the German institute IQWiG identified procedures and interventions for which possible overuse is intensively discussed.

5 In this context, indication means: The patients with the underlying diseases eligible for a second opinion according to the Directive typically did not receive an indication for surgery at their first consultation
