# Supplementary material for: Second opinion opportunity declined: patient typology and experiences regarding the decision-making process preceding elective surgeries in Germany
Source: BMC Health Serv Res. 2022 Nov 8;22:1329. doi: 10.1186/s12913-022-08742-4 (PMC9643974; doi:10.1186/s12913-022-08742-4)
Supplement: Supplementary file 1 — Additional file 1. Procedure of the SOD [file 12913_2022_8742_MOESM1_ESM.pdf]

Supplementary Material 1: Procedure of the SOD

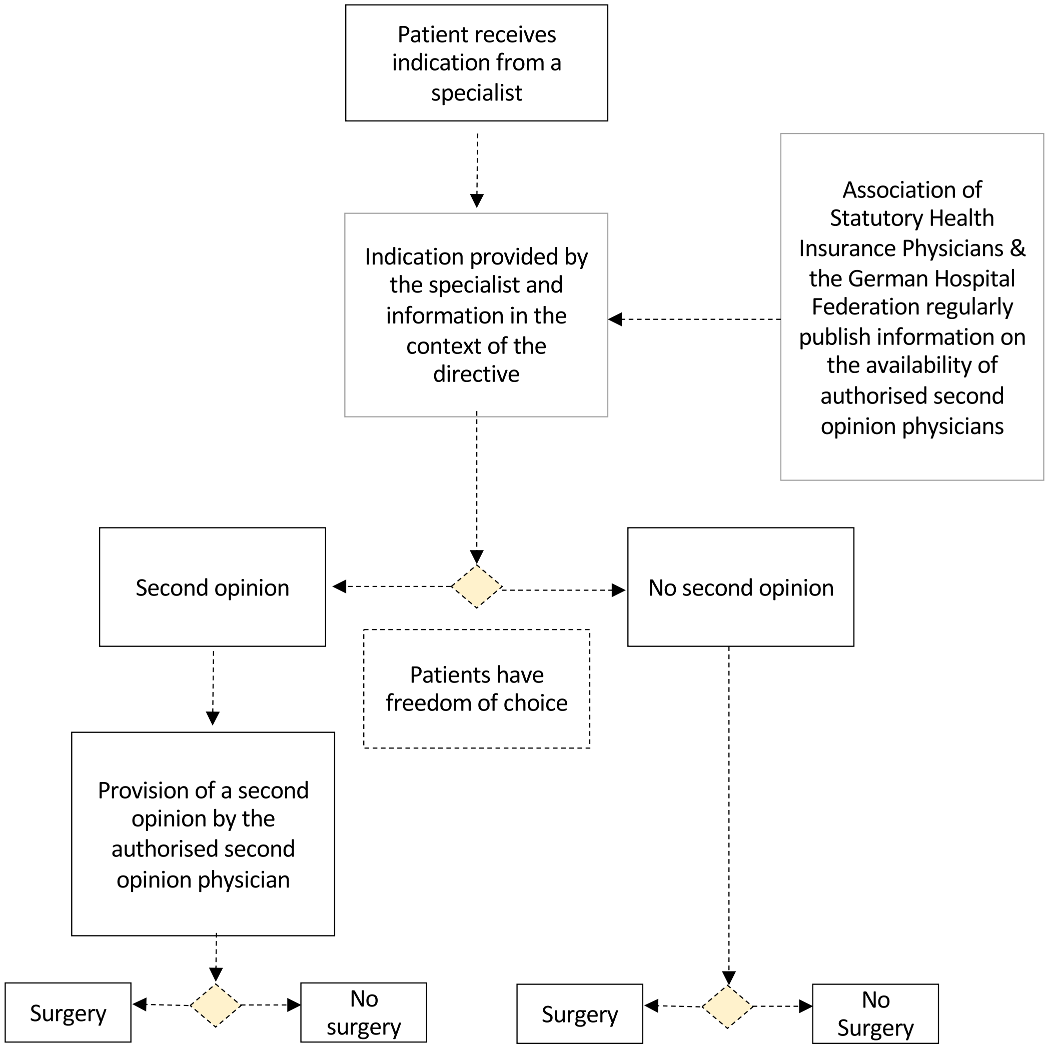

The physician who provides a recommendation for SOD-covered types of surgery must inform the patients at least ten days before planned surgery regarding the following items:
-her/his right to obtain a second opinion from an authorised second-opinion physician
-provision of information on available authorised secon-opinion physician
-informing that the second opinion cannot be provied by physician or intsitution designated to perform the procedure
-provision decision-making aids
-handing out a patient information sheet
-informing about the availability of medical report documents for the authorised second-opinion physician

Requirements for the second medical physician
-Specialised medical practice
-at least 5 years of full-time practice
-Special qualification: a post-doctoral lecture or an examiner-certification
-Information and counselling on possible alternative therapies or courses of action that an informed decision with regard to the necessity of performing the recommended intervention
