# Supplementary material for: Second opinion opportunity declined: patient typology and experiences regarding the decision-making process preceding elective surgeries in Germany
Source: BMC Health Serv Res. 2022 Nov 8;22:1329. doi: 10.1186/s12913-022-08742-4 (PMC9643974; doi:10.1186/s12913-022-08742-4)
Supplement: Supplementary file 2 — Additional file 2. Interview guide [file 12913_2022_8742_MOESM2_ESM.pdf]

Supplementary Material 2

Table 1. Interview guide

Guiding Questions	Checkaspects
How did you experience the treatment from diagnosis to surgery?<br><br>What did you feel during this time?<br><br>What feelings accompanied you?<br><br>How well were you informed about the consequences of the surgery?<br><br>How well were you informed about possible alternative treatment options / strategies (incl. Watchful Waiting) and their advantages and disadvantages?<br><br>How easy or difficult was it for you to assess the advantages and disadvantages of different treatment options?	Warm up<br><br>Experiencing the process of treatment<br><br>Process description
Did you consider getting a second opinion?<br><br>Did you get a second opinion?<br><br>If yes:<br>How did you experience the process? Or how did you go about getting a second opinion?<br><br>How did you go about choosing a second opinion?<br><br>What was your motivation for seeking a second opinion?<br><br>Did the first and second opinions concur?<br><br>Did the second opinion help you in reaching your decision?<br><br>Would you want to obtain a second opinion again?<br><br>If no:<br>Why did you not seek a second opinion?	Obtaining a second opinion
Can you please describe how you made the decision to have /undergo surgery?	Decision making process/ influencing factors

Would you please tell me when you made the decision to have your surgery?<br><br>What aspects played a role in your decision making for surgery?<br><br>What role did information-gathering play in your decision-making for surgery?<br><br>What role do other people play in making decisions?	
