# Supplementary material for: Second opinion opportunity declined: patient typology and experiences regarding the decision-making process preceding elective surgeries in Germany
Source: BMC Health Serv Res. 2022 Nov 8;22:1329. doi: 10.1186/s12913-022-08742-4 (PMC9643974; doi:10.1186/s12913-022-08742-4)
Supplement: Supplementary file 4 — Additional file 4. Detailed socio-demographic information [file 12913_2022_8742_MOESM4_ESM.pdf]

Supplementary Material 4: Detailed socio-demographic information

ID	Age	Sex	Settlement pattern	Educational level	Highest qualification	Surgery	Date Surgery	Decisional Type
MaG201_HE	32	female	Towns and suburbs (>5.000-100.000)	9–10 years of school completed	Apprenticeship	HE	2018	Type 4
MäG201_HE	45	female	Towns and suburbs (>5.000-100.000)	9–10 years of school completed	Apprenticeship	HE	2019	Type 1
MaG202_HE	47	female	Cities (>100.000)	9–10 years of school completed	Diplom	HE	2019	Type 3
MäG202_HE	80	female	Cities (>100.000)	9–10 years of school completed	Apprenticeship	SA	2019	Type 1
MäG203_SA	80	female	Cities (>100.000)	9–10 years of school completed	Apprenticeship	SA	2019	Type 1
MaG203_TE	36	male	Towns and suburbs (>5.000-100.000)	> 10 years of school completed	Apprenticeship	TE	2019	Type 3
MaG204_HE	55	female	Towns and suburbs (>5.000-100.000)	9–10 years of school completed	Apprenticeship	HE	2018	Type 4
MäG204_TE	34	male	Cities (>100.000)	9–10 years of school completed	Apprenticeship	TE	2019	Type 4
MaG205_HE	49	female	Cities (>100.000)	9–10 years of school completed	Apprenticeship	HE	2019	Type 3
MäG205_SA	59	female	Rural region (<5.000)	9–10 years of school completed	Apprenticeship	SA	2020	Type 1
MaG206_HE	50	female	Rural region (<5.000)	9–10 years of school completed	Apprenticeship	HE	2019	Type 1
MäG206_HE	76	female	Cities (>100.000)	9–10 years of school completed	Apprenticeship	HE	2019	Type 2
MaG207_SA	64	male	Rural region (<5.000)	9–10 years of school completed	Apprenticeship	SA	2020	Type 1
MäG207_SA	77	female	Cities (>100.000)	9–10 years of school completed	Apprenticeship	SA	2020	Type 3
MaG208_TE	23	male	Towns and suburbs (>5.000-100.000)	9–10 years of school completed	none	TE	2019	Type 3
MaG209_SA	65	male	Cities (>100.000)	9–10 years of school completed	Apprenticeship	SA	2018	Type 3
MäG209_TE	25	female	Cities (>100.000)	> 10 years of school completed	none	TE	2020	Type 4
MaG210_HE	43	female	Rural region (<5.000)	> 10 years of school completed	Apprenticeship	HE	2019	Type 3
MäG210_HE	44	female	Cities (>100.000)	9–10 years of school completed	Apprenticeship	HE	2019	Type 4
MaG211_TE	66	male	Cities (>100.000)	9–10 years of school completed	Apprenticeship	TE	2019	Type 3
MaG212_SA	60	female	Cities (>100.000)	9–10 years of school completed	Apprenticeship	SA	2018	Type 1
MäG212_SA	64	male	Cities (>100.000)	9–10 years of school completed	Apprenticeship	SA	2019	Type 1
MaG213_HE	48	female	Towns and suburbs (>5.000-100.000)	> 10 years of school completed	Apprenticeship	HE	2019	Type 1
MäG213_HE	51	female	Cities (>100.000)	9–10 years of school completed	Apprenticeship	HE	2019	Type 3
MaG214_SA	53	female	Rural region (<5.000)	9–10 years of school completed	Masters or Magisters degree/ Diploma	SA	2020	Type 4
MäG214_SA	35	male	Cities (>100.000)	> 10 years of school completed	Apprenticeship	SA	2020	Type 4
MaG215_TE	24	female	Cities (>100.000)	> 10 years of school completed	Masters or Magisters degree/ Diploma	TE	2018	Type 3
MaG216_HE	49	female	Cities (>100.000)	9–10 years of school completed	Apprenticeship	HE	2019	Type 3
MaG217_HE	59	female	Towns and suburbs (>5.000-100.000)	9–10 years of school completed	Apprenticeship	HE	2019	Type 3
MäG218_HE	69	female	Cities (>100.000)	9–10 years of school completed	Apprenticeship	HE	2020	Type 1
MaG218_SA	61	male	Cities (>100.000)	9–10 years of school completed	Apprenticeship	SA	2019	Type 1
MaG219_SA	69	male	Towns and suburbs (>5.000-100.000)	9–10 years of school completed	Apprenticeship	SA	2020	Type 1
MaG220_SA	61	female	Towns and suburbs (>5.000-100.000)	> 10 years of school completed	Masters or Magisters degree/ Diploma	SA	2019	Type 3
MäG220_SA	69	female	Rural region (<5.000)	9–10 years of school completed	Apprenticeship	SA	2020	Type 1
MaG221_HE	40	female	Cities (>100.000)	9–10 years of school completed	Apprenticeship	HE	2018	Type 4
MäG221_HE	43	female	Towns and suburbs (>5.000-100.000)	9–10 years of school completed	Apprenticeship	HE	2020	Type 3
MaG222_HE	85	female	Rural region (<5.000)	9–10 years of school completed	Apprenticeship	HE	2019	Type 2
MäG222_HE	46	female	Towns and suburbs (>5.000-100.000)	9–10 years of school completed	Apprenticeship	HE	2020	Type 4
MaG223_TE	55	male	Towns and suburbs (>5.000-100.000)	> 10 years of school completed	Apprenticeship	TE	2018	Type 4
MaG224_TE	35	female	Cities (>100.000)	9–10 years of school completed	Apprenticeship	TE	2018	Type 4
MaG225_SA	57	male	Cities (>100.000)	9–10 years of school completed	Bachelor	SA	2020	Type 4
MaG226_SA	57	male	Towns and suburbs (>5.000-100.000)	9–10 years of school completed	Apprenticeship	SA	2019	Type 1
MaG227_SA	42	male	Cities (>100.000)	9–10 years of school completed	none	SA	2019	Type 1

MaG228_HE	44	female	Cities (>100.000)	9–10 years of school completed	Bachelor	HE	2018	Type 2
MaG229_TE	29	male	Cities (>100.000)	> 10 years of school completed	Masters or Magisters degree/ Diploma	TE	2018	Type 3
MaG231_TE	42	female	Cities (>100.000)	9–10 years of school completed	Apprenticeship	TE	2018	Type 1
MaG232_SA	64	male	Cities (>100.000)	9–10 years of school completed	Apprenticeship	SA	2019	Type 3
MaG233_HE	54	female	Cities (>100.000)	> 10 years of school completed	Masters or Magisters degree/ Diploma	HE	2019	Type 4
MaG234_HE	68	female	Cities (>100.000)	9–10 years of school completed	Apprenticeship	HE	2018	Type 2
MaG235_SA	57	male	Towns and suburbs (>5.000-100.000)	No formal schooling	none	SA	2018	Type 3
MaG236_TE	28	female	Cities (>100.000)	9–10 years of school completed	Apprenticeship	TE	2018	Type 3
MaG237_TE	33	female	Cities (>100.000)	9–10 years of school completed	Apprenticeship	TE	2018	Type 1
MaG238_SA	55	female	Cities (>100.000)	9–10 years of school completed	Apprenticeship	SA	2018	Type 1
MaG239_TE	20	male	Towns and suburbs (>5.000-100.000)	No formal schooling	none	TE	2018	Type 3
MaG240_TE	23	female	Cities (>100.000)	> 10 years of school completed	Apprenticeship	TE	2018	Type 3
MaG241_HE	46	female	Cities (>100.000)	> 10 years of school completed	Apprenticeship	HE	2018	Type 1
MaG242_TE	50	female	Towns and suburbs (>5.000-100.000)	9–10 years of school completed	Apprenticeship	TE	2018	Type 3
MaG243_SA	67	female	Towns and suburbs (>5.000-100.000)	9–10 years of school completed	Apprenticeship	SA	2018	Type 1
MaG244_HE	36	female	Rural region (<5.000)	> 10 years of school completed	Apprenticeship	HE	2018	Type 1
MaG245_TE	38	male	Cities (>100.000)	> 10 years of school completed	Masters or Magisters degree/ Diploma	TE	2018	Type 3
MaG246_TE	36	female	Cities (>100.000)	9–10 years of school completed	Apprenticeship	TE	2017	Type 4
MaG247_TE	26	female	Cities (>100.000)	> 10 years of school completed	Apprenticeship	TE	2018	Type 4
